# Artesunate-mefloquine combination therapy in acute *Plasmodium falciparum *malaria in young children: a field study regarding neurological and neuropsychiatric safety

**DOI:** 10.1186/1475-2875-9-291

**Published:** 2010-10-21

**Authors:** Sarabel G Frey, David Chelo, Mina N Kinkela, Florence Djoukoue, Felix Tietche, Christoph Hatz, Peter Weber

**Affiliations:** 1 Division of Neuropaediatrics and Developmental Medicine, University Children's Hospital Basel, Switzerland; 2 Centre Mère et Enfant, Fondation Chantal Biya, Yaoundé, Cameroon; 3 Swiss Tropical and Public Health Institute, Basel, and University of Zürich, Switzerland

## Abstract

**Background:**

Mefloquine-artesunate combination therapy for uncomplicated falciparum malaria is one of the treatments used in African children. Data concerning neurological safety in adults and children treated with mefloquine and artesunate combination therapy is well documented in Asia. Safety data for neurological and neuropsychiatric side effects of mefloquine and artesunate combination therapy in African children are scarce, although WHO recommends this therapy in Africa.

**Methods:**

A phase IV, open label, single arm study was conducted among African children between 10 and 20 kg with acute uncomplicated falciparum malaria. They were treated over three consecutive days with a paediatric fixed-dose combination of artesunate (50 mg/d) and mefloquine (125 mg/d). Parasitological, clinical and neurological examinations and standardized questions about neuropsychiatric symptoms were carried out on days 0, 4, 7, 28 and 63. The primary objective was to assess the neurological and neuropsychiatric safety of artesunate-mefloquine combination therapy in young children.

**Results:**

From December 2007 to March 2009, 220 children with uncomplicated *Plasmodium falciparum *malaria were treated with artesunate and mefloquine. 213 children were analysed according to study protocol. 50 neurological and neuropsychiatric adverse events occurred in 28 patients. Eleven drug-related neurological and neuropsychiatric adverse events occurred in eight patients. Sleeping disorders were present in 2.3%, neurological disorders in 1.4%, neuropsychiatric disorders in 1% and eating disorders in 0.5% of the patients. Adverse events were of mild to moderate intensity and resolved spontaneously.

**Conclusion:**

African children showed a low percentage of self-limited neurological and neuropsychiatric adverse events, confirming studies on neurological safety in Asian children treated with artesunate and mefloquine. Sleeping disorders were most frequently observed.

## Background

Malaria is an important cause of infant morbidity and mortality in African children, especially in young children who lack immunity. According to the World Health Organization (WHO), 36% of African children of <5 years receive anti-malarial treatment. The infant mortality rate (< 5 y) in Africa is 145/1000 live births/year, of which 15.6% are caused by malaria (= 22,6/1000 live births/y). In some African countries infant mortality caused by malaria is as high as 29.7% [1].

Effective treatment is very important and has become more difficult due to emerging drug resistance. Since 2006 the WHO recommends artesunate plus amodiaquine, artesunate plus lumefantrine and artesunate plus sulphadoxine/pyrimethamine as first-line treatment for uncomplicated *Plasmodium falciparum *malaria in Africa. Artemisinin-based combination therapy has proven to be highly effective and safe. A rapid clearance of parasitaemia and a rapid solution of clinical symptoms have been shown in several clinical trials and in every day use. In Asia artesunate plus mefloquine is recommended by the World Health Organization for children and adults since 2006. In 2010, WHO released new recommendations, which include artesunate plus mefloquine therapy for uncomplicated *P. falciparum *malaria in Africa. This combination is well tolerated in every day use and shows excellent efficacy combined with a good safety profile in several clinical trials [1-4].

In adults, mefloquine is known to show, especially in long-term prophylaxis, a variety of rare neurological/neuropsychiatric adverse effects, for example headache, vertigo, insomnia, depression, anxiety, sensory and motor neuropathy, tinnitus, reversible hypoacusis and myopathy. The elimination half-life of mefloquine varies from 14 to 41 days and sub-therapeutic concentrations persist in the body for several months [5].

No data regarding specific neurological/neuropsychiatric adverse effects of mefloquine combination therapy with artesunate for African children has been published, whereas data for Asian children are available [2,3]. The presence and quality of neurological/neuropsychiatric adverse effects may not have been reported because they would not have been recognized due to the young age of the children. It is possible that parents do not associate the neuropsychiatric or neurological side effects with the treatment of malaria, especially with respect to the delay between the intake and the occurrence (mean half-life of mefloquine is 21d). A study in Asian children regarding neurological safety after use of mefloquine and mefloquine-artesunate combination therapy in uncomplicated falciparum malaria showed no significant decrease in neurological performance in comparison to a control group [1]. This study aimed at quantifying efficacy and neurological and neuropsychiatric safety in a three-day artesunate-mefloquine combination therapy of acute falciparum malaria during everyday use in infants and young children in Africa.

## Methods

The clinical trial protocol was approved by the national Ethic Committee of Yaoundé, Cameroon, and by the Ethics Committee of the Technical University of Dresden, Medical Faculty, Germany. This study is registered with ClinicalTrials.gov as NCT00978172.

### Study population

Young children between 10 and 20 kg of weight attending the Clinic of *Centre Mère et Enfant de la Fondation Chantal Biya *in Yaoundé, Cameroon, were considered for enrolment if they presented with acute uncomplicated *P. falciparum *malaria (according to WHO criteria). Inclusion criteria: count of asexual forms of *P. falciparum *between 2,000 (amended to 1,000 μl of blood) and 250,000 parasites, fever or history of fever, written informed consent of the guardian, and the ability of the child to take oral medication.

Exclusion criteria: children were excluded if they suffered from severe malaria (according to WHO criteria), or had a history or evidence of clinically significant neurological, psychiatric, cardiovascular, pulmonary, metabolic, gastrointestinal, oncologic or endocrine disease. In addition, children were excluded if they were treated with anti-malarial drugs within 7 days prior to diagnosis or if they had participated in any investigational drug trial within 30 days prior to enrolment. Further exclusion criteria were vomiting three or more times within 24 h of enrolment, more than three copious liquid stools within 24 h, parenteral treatment, allergy to artesunate or mefloquine, splenectomy, HIV infection, or renal impairment.

### Protocol

A full medical history and clinical examination was carried out after obtaining consent from the children's guardians. The neurological and neuropsychiatric assessment was performed before administration of the study drug and presented the baseline value.

According to the study protocol, examination visits took place on baseline, and days 4, 7 (±1), 28 (±2) and 63 (±4). Malaria blood smears were checked on each visit and PCR blood samples were taken at each visit. Haematological exams (haemoglobin, haematocrit, RBC, WBC) were carried out on baseline and days 4, 7, 28 and 63. A complete physical examination was carried out on baseline and day 63. Vital signs and symptoms of malaria were recorded during every visit. Adverse events could be reported any time, at the latest on the next visit.

Adverse events were defined and classified as "mild", "moderate" and "severe" according to ICH Harmonised Tripartite Guideline for Good Clinical Practice E6 (R1) [6]. All adverse events were recorded. For evaluation they were divided into "drug-related" ("certain", "probably", "possibly" and "unlikely") and "not drug-related". A written statement by the investigator was required to justify why the neurological and neuropsychiatric adverse event was not drug-related.

### Anti-malarial drug treatment

Anti-malarial treatment was given over three consecutive days with a daily oral dose of Artequin^® ^Paediatric (Stick pack) containing 50 mg of artesunate and 125 mg of mefloquine in a fixed combination. The first dose was given to the child by the investigator in the health centre, the second and third doses by the guardian of the child at home. If vomiting occurred within the first 30 minutes after intake, a full replacement dose (one stick pack) was given. Replacement was possible only once. The guardians had to return empty stick packs on day 4 to the investigator in order to verify compliance with the treatment schedule. The Study medication was provided by Mepha Ltd, Aesch, Switzerland.

### The neurological and neuropsychiatric examination

The neurological and neuropsychiatric examination took place on the baseline visit and on days 7, 28 and 63. Performing the examination took approximately 30 minutes. Children were questioned and examined by the same investigator during all visits. Three experienced paediatricians of the *Centre Mère et Enfant de la Foundation Chantal Biya *were trained in the use of the questionnaire and in the standardized neurological assessment with a video recording of the examination and by examining volunteer patients according to the protocol. This training involved demonstrating the tests and then getting the investigator to perform and score the examination by consensus. During the study course, the training was repeated after 7 months.

Phrasing and questions were tested in about 15 patients and guardians in Yaounde, Cameroon prior to the beginning of the study to ensure, that the questions were well understood by the guardians to identify accurately the neuropsychological symptoms.

The neurological and neuropsychiatric examination included 23 questions to the guardian concerning his/her observations, questions to the child (older than 3.5 y as a rule), the investigators observations and twelve clinical assessments. The questionnaire was available in French and English, containing twenty-three prephrased questions covering dizziness, vertigo, headache, convulsions, paraesthesia (only >3.5 y), dysaesthesia (only >3.5 y), insomnia, nightmares (only >5 y), hyperactivity, anxiety, panic attacks, sadness, mood changes, confusion, aggressive behaviour, tension, visual hallucinations, acoustic hallucinations (only >3.5 y), hearing loss, dysarthria, word-finding disturbance (only >3.5 y), eating behaviour and swallowing disturbance. Questions were addressed to the guardian with specific questions to the child him/herself. To avoid misunderstanding or misinterpretation, all investigators used the same terms to ask those questions.

The neurological examination covered twelve items: tremor, dystonia and ataxia, hyperreflexia, hyporeflexia, clonus, dysdiadochokinesis, disturbed vision, nystagmus, double vision, acoustic acuity, forgetfulness, and word-finding disturbance. For this exam investigators were equipped with an examination set containing a description of the examination process, a reflex hammer, a lamp and a *Lang Stereo Vision *test (item disturbed vision), five coloured building blocks (item forgetfulness), a toy car (item word-finding disturbance), and a *Denver Developmental *test.

Tremor, dystonia and ataxia, hyperreflexia, hyporeflexia, clonus, dysdiadochokinesis, nystagmus, double vision and acoustic acuity were tested doing a standard paediatric neurological examination based on Touwen [7]. Word-finding disturbance was tested by showing the child a toy car, a bird (or telephone), a key, a pen, a chair and asking to name the object. A score of "NOT present" for word-finding disturbance was given if the child could name all objects promptly.

Some exams/questions were age restricted to certain age limits or modified according to the age of the child. Item forgetfulness was tested in children younger than 3.5 years by presenting the child 3 coloured building blocks in a given order and letting the child repeat the placement after mixing the building blocks. Forgetfulness in children older than 3.5 years was tested by enumerating number groups. To begin the test, the child had to repeat two groups of two digits, then two groups of three digits, then two groups of four digits, then two groups of five digits.

During the baseline visit, it was recorded whether a 2-digit, 3-digit, 4-digit or 5-digit group could be repeated, and a score of one point per correct repeat was given. During follow-up visits, the forgetfulness was recorded as "present" if there was a decrease in the digit group that could be repeated. For example, if a child was able at baseline to repeat a 4-digit group but could not repeat a 3-digit group on day 28, this was marked as presence of forgetfulness. This procedure was chosen in accordance with tasks from cognitive tests such as Hamburg Wechsler Intelligence Test for Children [8] or Kaufman-Assessment Battery for Children [9].

Children could refuse to participate. If the exam could not be performed investigators scored this with "not done". Otherwise questions were scored with "yes (disorder present) " or "no (disorder not present) " at baseline. In the follow-up visits questions were scored with "no (disorder not present) " or "yes (disorder present, still the same)" or "yes (disorder present, improved)" or "yes (disorder present, worsened or new)" in which case an adverse event was reported. The option "not done" was chosen if the child was not cooperating. All children were followed up for fever and parasite clearance irrespective of their participation in neurological and neuropsychiatric examinations.

Only the appearance of new symptoms - occurring after baseline - was evaluated as an adverse event. If a child was already hyperactive before taking the study medication, this was not considered as an adverse event at the next study visit, if it was still present. But if hyperactivity appeared newly afterwards during the study course or worsened during two visits, it was reported as an adverse event.

The clinical neurological and neuropsychiatric examination is based on standard paediatric neurological examination (based on Touwen [7]); the questionnaire was adapted to symptoms recorded in adults during mefloquine treatment or prophylaxis. With respect to the absence of a standardized test for paediatric neurological and neuropsychiatric evaluation in this age group in a resource-poor setting, the Division of Child Neurology of the University Children's Hospital of Basel, Switzerland, developed the examination setting used in this study.

### Laboratory procedure

Laboratory procedures have been described elsewhere [10].

### Concomitant medication

Concomitant medication was recorded; children taking other malaria treatments due to a new malaria episode completed all visits up to day 63, but evaluation for neurological and neuropsychiatric events was only validated until the day beginning the concomitant malaria medication.

### Statistical methods for evaluating neurological and neuropsychiatric safety

Only children who took at least one dose of study medication and who had the neuropsychiatric and neurological exam at least at baseline and day 7 or had a drug-related neurological or neuropsychiatric adverse event were included in the statistical evaluation of neuropsychiatric and neurological safety. The proportion of adverse events was calculated as well as the confidence interval.

## Results

### Study participants

From December 2007 to March 2009, 220 children with uncomplicated *P. falciparum *malaria were included in the study, of which 184 children (83.6%) concluded the study according to protocol. 3,800 children had been screened (including blood smear), 220 met the inclusion criteria; 94% were not included because of negative blood smear or parasite count less than 2,000 (amended to 1,000 μl of blood) or more than 250,000. Data from 213 patients were analysed for neuropsychiatric and neurological safety. Two patients were classified as early treatment failure; five patients had no follow-up visits and were not evaluable for neuropsychiatric and neurological safety.

The youngest child included was seven months old; the oldest seven years and nine months. The mean age was three years and four months. (27 children were younger than 1.5 years, 104 children were between 1.5 and 3.5 years, 51 children were 3.5 to 5 years old and 31 children were older than 5 years). Gender distribution was equal: 104 boys (48.8%) and 109 girls (51.2%) participated in the study.

### Neurological and neuropsychiatric outcome (safety)

**Figure 1:**
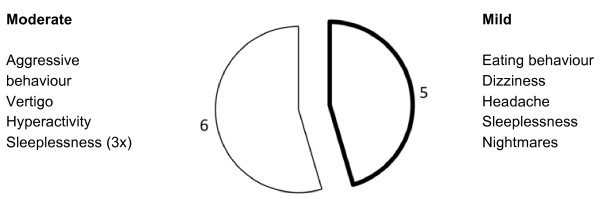
**Number and intensity of drug related N+N adverse events**.

Fifty neuropsychiatric and neurological adverse events occurred in 28 children (13%). Eleven neuropsychiatric and neurological adverse events in eight children (out of 213; 5.16%) were related to the study medication. (Table 1 and Figure 1). The frequency of the occurrence of at least one neuropsychiatric and neurological adverse event related to the study medication was 3.77%, (95% CI 1.6-7.3%). The age distribution of drug-related neuropsychiatric and neurological adverse events did not reveal any differences (age group <1.5 years 3.7%, 1.5-3.5 years: <3.8%, 3.5-5 years: 3.9%, >5 years: 3.7%).

**Table 1:** Occurrence of drug-related neuropsychiatric and neurological adverse events

Neuropsychological or neurological symptom	Number of patients suffering from at least one event/number of analyzed patients*	95-% confidence interval
**Dizziness**	1/213	0.0 - 2.59%

**Headache**	1/209	0.0 - 2.64%

**Vertigo**	1/211	0.0 - 2.61%

**Convulsions**	0/212	0.0 - 1.72%

**Paraesthesia**	0/76	0.0 - 4.74%

**Dysaethesia**	0/76	0.0 - 4.74%

**Sleeplessness (insomnia)**	4/213	0.5 - 4.74%

**Nightmares**	1/30	0.1 - 17.2%

**Hyperactivity**	1/213	0.0 - 2.59%

**Anxiety**	0/213	0.0 - 1.72%

**Panic Attack**	0/213	0.0 - 1.72%

**Unexplained sadness**	0/213	0.0 - 1.72%

**Mood changes**	0/213	0.0 - 1.72%

**Confusion**	0/212	0.0 - 1.72%

**Aggressive behavior**	1/213	0.0 - 2.59%

**Tension**	0/213	0.0 - 1.72%

**Visual hallucinations**	0/80	0.0 - 4.51%

**Acoustic hallucinations**	0/76	0.0 - 4.74%

**Hearing loss**	0/211	0.0 - 1.73%

**Dysarthria**	0/209	0.0 - 1.75%

**Word finding disturbance (1)**	0/204	0.0 - 1.79%

**Eating behavior**	1/213	0.0 - 2.59%

**Swallowing disturbance**	0/213	0.0 - 1.72%

**Tremor**	0/211	0.0 - 1.73%

**Dystonia, ataxia**	0/212	0.0 - 1.72%

**Hypereflexia**	0/213	0.0 - 1.72%

**Hyporeflexia**	0/213	0.0 - 1.72%

**Clonus**	0/213	0.0 - 1.72%

**Dysdiadochokinesis**	0/164	0.0 - 2.22%

**Disturbed vision**	0/151	0.0 - 2.41%

**Nystagmus**	0/199	0.0 - 1.84%

**Double vision**	0/71	0.0 - 5.06%

**Acoustic acuity**	0/122	0.0 - 2.98%

**Forgetfullness**	0/93	0.0 - 3.89%

**Word finding disturbance (2)**	0/72	0.0 - 4.99%

Nightmares were only assessed in children 5 years in age or older

Paraesthesia, dysaethesia, visual and acoustic hallucinations, double vision and word finding disturbance (2) were only assessed in children 3.5 years in age or older

Acoustic acuity was only assessed in children 1.5 years in age and or older

* N = Number of patients who had the examination at Day 7 or for whom the related NNAE was reported at any time

**Figure 2:**
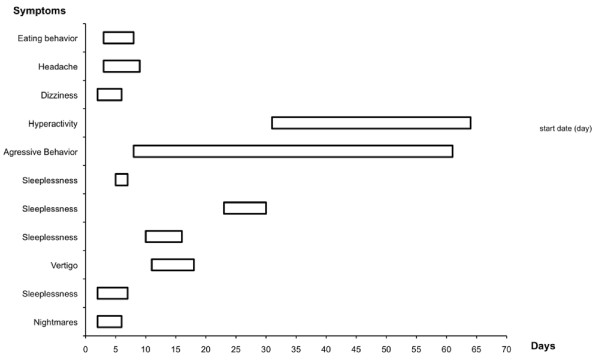
**Onset and duration of 11 drug related N+N adverse events (which occurred in 8 patients)**.

Results of the registration of neurological and neuropsychiatric disorders at baseline are shown in Table 2. At baseline, hyporeflexia or dystonia (hypotonia) were present in 5.4% of the children. At baseline, altered eating behaviour was present in 72% of the children. During the study reported drug-related neurological and neuropsychiatric adverse events were of mild (45.5%) or moderate (54.5%) intensity. Onset of the drug-related adverse events occurred during the first five days after baseline in 45% of the children, an additional 36% occurred between days 5 and 15. The latest adverse event was recorded on day 31. Resolution of the adverse events occurred 10 days after onset in 81% of the adverse events. The resolution of one adverse event (moderate hyperactivity) was not clear, because of insufficient follow-up; all other adverse events were resolved spontaneously (Figure 2).

**Table 2:** Neuropsychiatric and neurological disorder examination at baseline

Symptom	Yes		No		ND	
	
	N	%	N	%	N	%
**Dizziness**	93	43.7	120	56.3	.	.

**Headache**	85	39.9	114	53.5	14	6.6

**Vertigo**	14	6.6	191	89.7	8	3.8

**Convulsions**	.	.	213	100.0	.	.

**Paraesthesia**	1	1.2	77	93.9	4	4.9

**Dysaethesia**	.	.	78	95.1	4	4.9

**Sleeplessness (insomnia)**	85	39.9	128	60.1	.	.

**Nightmares**	2	6.5	28	90.3	1	3.2

**Hyperactivity**	18	8.5	195	91.5	.	.

**Anxiety**	26	12.2	187	87.8	.	.

**Panic Attack**	30	14.1	183	85.9	.	.

**Unexplained sadness**	23	10.8	190	89.2	.	.

**Mood changes**	25	11.7	188	88.3	.	.

**Confusion**	4	1.9	208	97.7	1	0.5

**Aggressive behavior**	19	8.9	194	91.1	.	.

**Tension**	12	5.6	201	94.4	.	.

**Visual hallucinations**	3	3.7	77	93.9	2	2.4

**Acoustic hallucinations**	.	.	78	95.1	4	4.9

**Hearing loss**	.	.	211	99.1	2	0.9

**Dysarthria**	3	1.4	205	96.2	5	2.3

**Word finding disturbance (1)**	.	.	200	93.9	13	6.1

**Eating behavior**	154	72.3	59	27.7	.	.

**Swallowing disturbance**	.	.	213	100.0	.	.

**Tremor**	.	.	205	96.2	8	3.8

**Dystonia, ataxia**	3	1.4	209	98.1	1	0.5

**Hypereflexia**	.	.	213	100.0	.	.

**Hyporeflexia**	8	3.8	205	96.2	.	.

**Clonus**	.	.	213	100.0	.	.

**Dysdiadochokinesis**	1	0.5	137	64.3	75	35.2

**Disturbed vision**	.	.	128	60.1	85	39.9

**Nystagmus**	.	.	192	90.1	21	9.9

**Double vision**	1	1.2	62	75.6	19	23.2

**Acoustic acuity**	1	0.5	100	53.8	85	45.7

**Forgetfullness**	1	0.5	70	32.9	142	66.7

**Word finding disturbance (2)**	1	1.2	68	82.9	13	15.9

Nightmares were only assessed in children 5 years in age or older

Paraesthesia, dysaethesia, visual and acoustic hallucinations, double vision and word finding disturbance (2) were only assessed in children 3.5 years in age or older

Acoustic acuity was only assessed in children 1.5 years in age and or older

The most common drug-related adverse events were sleeping disorders (2.3%), insomnia (4/213 children, 1.9%) and nightmares (1/31 children 3.2%). Neurological disorders were present in three children (1.4%): vertigo (0.5%), dizziness (0.5%), headache (0.5%). Neuropsychiatric disorders (1%) were present in one child who had two adverse events: hyperactivity (0.5%) and aggressive behaviour (0.5%). Abnormal eating behaviour (0.5%) was present in one other child.

One serious neurological and neuropsychiatric adverse event, which was classified as not drug-related, occurred. The child was suffering from convulsions during the night after baseline and was hospitalized. The convulsions were attributed to the underlying disease (malaria), and resolution under different malaria medication was achieved without complications.

### Non-neurological and neuropsychiatric outcome (safety)

There were five serious adverse events in four children during 63 days of follow-up. One child was hospitalized twice because of anaemia, two children were hospitalized because of severe malaria, and one child was hospitalized because of asthenia. All were classified to be not drug-related. A total of 169 non-neurological and neuropsychiatric adverse events in 112 patients have been reported. Eleven of the 169 other adverse events were classified as related to the study drug. The most frequently related adverse event was anaemia (6 children 2.8%), followed by diarrhoea (2 children 0.9%), vomiting (2 children 0.9%) and sleep disorder (1 patient 0.5%). All of the related adverse events were mild or moderate in intensity. Precise description and discussion is published elsewhere [10].

### Parasitological outcome (efficacy)

PCR corrected ACPR (Adequate clinical and parasitological response) rates were 96.6% (95% CI: 93.0 to 98.4%) for day 28 and for day 63. Four patients were classified as late clinical failures and three patients as early treatment failures. There were 22 new infections up to day 63 which could be confirmed as new malaria episodes by PCR. Median time for the children who were suffering from a new infection was 56 days. More detailed information about the parasitological outcome is published elsewhere [10].

### Concomitant medication

75.3% of all participating children were treated with analgesics as concomitant medication during the study course. 51.2% of the children took anti-anaemic medication. 18.6% of the children were treated with anti-inflammatory and anti-rheumatic medication. 8.4% of the children were treated with anti-helmetic medication. Cough and cold preparations were given to 7.4% of all children included in the study. 7.7% of the included children were treated with other medications. Duration of concomitant medications varied.

## Discussion

Detailed neurological and neuropsychiatric safety evaluation among African children aged 7 months to 7 years was performed after artesunate-mefloquine treatment of acute *P. falciparum *malaria. Patients from a district hospital represented the diversity of the population though the study was a single centre study. The large number (94%) of children screened but not included represents the reality in African cities where the clinical criteria of malaria often do not correspond with parasitaemia (parasite count in the blood smear). This leads to a large number of un-needed applications of anti-malarial therapy and aids the development of resistance against anti-malarial therapy.

One of the advantages of this study was to evaluate a large study population (213 children) under every day conditions. Study results show a very good efficacy [10], and neurological and neuropsychiatric safety for the paediatric fixed-dose combination artesunate-mefloquine.

Clinical neurological performance and neuropsychiatric status did not deteriorate (after treatment) in 96.2% of the children during the study. 3.8% of the children had a transient drug-related mild to moderate neurological or neuropsychiatric impairment, which resolved spontaneously.

Sleeping disorders were the most frequently observed neurological and neuropsychiatric adverse events. This confirms results of other studies conducted for safety in mefloquine or combined mefloquine-artesunate therapy in adults and children as malaria chemoprophylaxis or for treatment of uncomplicated falciparum malaria, where sleeping disturbance was a common adverse event [11,12]. An interesting point is that the adverse events (abnormal eating behaviour, headache, dizziness, sleeplessness and nightmares), which were reported by parents during the first five days of the study, were all disorders that could be attributed to the acute malaria episode.

Dizziness was the most common adverse event in other studies [13]. Dizziness often occurred at baseline, but only in one child (0.5%) during the post-treatment observation period. Aggressive behaviour or hyperactivity as adverse events were previously not reported among children after mefloquine-artesunate therapy in uncomplicated acute falciparum malaria. Both neuropsychiatric adverse events occurred in the same child (0.5% for each adverse event) and were most likely attributed to mefloquine. Case reports in children and studies in adults treated with mefloquine monotherapy or mefloquine combination therapy for prophylaxis or acute therapy [12,14], describe rare cases of mania, psychotic episodes, and anxiety attacks. Such events were not observed in the present study.

To date, only one study [2] has focused on CNS effects in young children with artesunate-mefloquine combination therapy. This study was conducted from 1994 to 1997 in ninety-eight Asian children and used a specified neurological assessment for children (Shoklo Neurological Test and Shoklo Developmental Test [15]). Repeated assessments in children aged three months to five years were conducted until day 28 after first intake of mefloquine-artesunate therapy. 45 children received an artesunate mono-therapy with a total dose of 12 mg/kg over 6 days, 46 children received an artesunate (12 mg/kg total dose) + mefloquine (25 mg/kg total dose) combination therapy over 8 days. 36 children were healthy control subjects.

The neurological assessment included evaluation of tonus, coordination, the status of consciousness, the emotional status and the social orientation [15]. In accordance with the presented data, this study showed no deterioration or deficits in coordination and tonus due to mefloquine-artesunate therapy, the other parameters showed no deterioration either, but were not directly comparable to items in our study.

A large sample (213 children) of a concise age group of children (7 month to 7 years) weighing 10-20 kilograms was investigated. Neuropsychiatric and neurological disorders were accurately recorded by a specific standardized questionnaire containing 23 questions in the present study. Tone and coordination disorders were evaluated in the same way as the other neurological disorders by the investigator in the clinical neurological exam as "present" or "not present" with only two items, which had to be quantified. Questions and neurological exams were age-adapted regarding the investigated symptom. For most questions and exams the investigation was different for children younger and older than 3.5 years. Questions and examinations were adapted to the applicability of the examination to the developmental status of the age of the children: for example nightmares were only applicable in children older than 5 years, because it was not possible to identify a nightmare in younger children according to their developmental status.

Because of the long half-life of mefloquine with measurable blood levels of mefloquine up to 41 days [16], neurological and neuropsychiatric assessments were done until day 63. Thus, late onset neurological and neuropsychiatric adverse events were recorded in contrast to the study by Ambler *et al *[2]. Hyperactivity, as the only neuropsychiatric adverse event (0.5%), was detected after day 28.

Standardized tests for detecting deficits in neuromotoric skills and abnormal neuropsychiatric features for children in this age group exist [7-9,17,18] but are not feasible in resource-poor settings, because they need expensive materials and are very time consuming. Therefore a feasible and age-adapted examination tool was established for this study. In comparison to the tool developed earlier by Haataja [15], with this new examination tool the investigation includes neuropsychiatric features. The detection of neuropsychiatric features was a main focus because of the known neuropsychiatric adverse effects of mefloquine in adults [13,19,20]. It is important to validate these examination tools further to provide in the future a more valid instrument of neuropsychiatric examination among children, especially of a young age.

The absence of a double testing excludes the possibility to measure an interrater reliability. The neurological and neuropsychiatric safety of the artesunate-mefloquine combination therapy in young children was the endpoint of the study. Therefore a standardized examination and questionnaire by the investigator were assumed to be sufficient. Precise acoumetry could not be performed, and therefore no new results to the present knowledge on hearing impairment related to artemisinin [21-23] could be added.

The initial assessment of the neurological status of young children in an acute malaria attack is very difficult. Children are often not cooperative during the acute phase of malaria and *P. falciparum *infection is likely to negatively influence CNS function [24]. This was reflected in the observation that one item of the neurological assessment (forgetfulness) at baseline was not completed by 66.7% of the children in the applicable age group. Three other neurological investigation items (dysdiadochokinesis, disturbed vision, acoustic acuity) could not be completed among 35-45% of the children in the applicable age group. When a baseline assessment was not possible, the follow-up assessments could not be compared to baseline and therefore an adverse event could not be properly assessed because it was not clear whether the disorder was already present at baseline. However, the results of the study are comparable to the study in Asian children [2]. Cooperation in a few children in this investigation remained difficult and sometimes was even impossible after the acute malaria symptoms had disappeared. Another limitation of this study is that there is no control group of healthy children. However, the intra subject design compensates this limitation.

Concomitant medication can influence neurological and neuropsychiatric examination. 73.5% of the children received analgesic medication; more than 50% took anti-anaemic medication. These results show that this study is a field study, where the safety of the mefloquine-artesunate therapy was evaluated in every day use. Less than 4% showed drug-related neurological and neuropsychiatric adverse events despite the fact that three-quarters of all children had taken concomitant medication, resulting in potential additional drug interactions and adverse events.

## Conclusion

The first assessment of neurological and neuropsychiatric safety of artesunate-mefloquine combination therapy in 213 African children with acute *P. falciparum *malaria and artesunate-mefloquine combination therapy over a period of 63 days showed no serious drug-related neurological or neuropsychiatric events. Less than 4% showed mild to moderate transient drug-related neurological or neuropsychiatric disorders, and most of them sleeping disorders. This study does not entirely exclude subtle or rare CNS effects of artesunate or mefloquine treatment, but underlines the neurological and neuropsychiatric safety of the paediatric fixed-dose combination of artesunate and mefloquine in young African children treated with artesunate-mefloquine for acute falciparum malaria.

## Conflicting interests

The authors declare that they have no conflict of interests. The study was sponsored by MEPHA LTD, Aesch, Switzerland

## Authors' contributions

FT was principal investigator and responsible for conducting the study. DC, FD and KM were investigators performing all clinical examinations. WP and FS developed the method for neurological and neuropsychiatric assessment. CH acted as a medical consultant. All authors read and approved the final manuscript.
